# ORENZA: a web resource for studying ORphan ENZyme activities

**DOI:** 10.1186/1471-2105-7-436

**Published:** 2006-10-06

**Authors:** Olivier Lespinet, Bernard Labedan

**Affiliations:** 1 Institut de Génétique et Microbiologie, CNRS UMR 8621, Université Paris-Sud, Bâtiment 400, 91405 Orsay Cedex, France

## Abstract

**Background:**

Despite the current availability of several hundreds of thousands of amino acid sequences, more than 36% of the enzyme activities (EC numbers) defined by the Nomenclature Committee of the International Union of Biochemistry and Molecular Biology (NC-IUBMB) are not associated with any amino acid sequence in major public databases. This wide gap separating knowledge of biochemical function and sequence information is found for nearly all classes of enzymes. Thus, there is an urgent need to explore these sequence-less EC numbers, in order to progressively close this gap.

**Description:**

We designed ORENZA, a PostgreSQL database of ORphan ENZyme Activities, to collate information about the EC numbers defined by the NC-IUBMB with specific emphasis on orphan enzyme activities. Complete lists of all EC numbers and of orphan EC numbers are available and will be periodically updated. ORENZA allows one to browse the complete list of EC numbers or the subset associated with orphan enzymes or to query a specific EC number, an enzyme name or a species name for those interested in particular organisms. It is possible to search ORENZA for the different biochemical properties of the defined enzymes, the metabolic pathways in which they participate, the taxonomic data of the organisms whose genomes encode them, and many other features. The association of an enzyme activity with an amino acid sequence is clearly underlined, making it easy to identify at once the orphan enzyme activities. Interactive publishing of suggestions by the community would provide expert evidence for re-annotation of orphan EC numbers in public databases.

**Conclusion:**

ORENZA is a Web resource designed to progressively bridge the unwanted gap between function (enzyme activities) and sequence (dataset present in public databases). ORENZA should increase interactions between communities of biochemists and of genomicists. This is expected to reduce the number of orphan enzyme activities by allocating gene sequences to the relevant enzymes.

## Background

Since 1956, the Nomenclature Committee of the International Union of Biochemistry and Molecular Biology (NC-IUBMB) has been classifying enzyme activities (EC numbers) in order to organize all contributions made by individual biochemists and to check their validity and consistency [1]. Such a standardization effort is based on the definition of the so-called EC numbers that comprise four digits. The first one (from 1 to 6) delineates the broad type of activity: Oxidoreductase, Transferase, Hydrolase, Lyase, Isomerase, and Ligase respectively. The second and third digits detail the reaction that an enzyme catalyzes. For example (Table 1), among the 1065 items forming the class Hydrolases (EC 3), there are 163 Glycosylases forming the subclass EC 3.2, of which, 140 enzymes hydrolyse O- and S-glycosyl compounds (sub-subclass EC 3.2.1) and 23 hydrolyse N-Glycosyl compounds (sub-subclass EC 3.2.2). The last digit is a serial number that is used to identify a particular enzyme. For instance, EC 3.2.2.1 corresponds to the purine nucleosidase and EC 3.2.2.3 to the uridine nucleosidase, respectively. The EC categorization is constantly evolving as new enzyme activities are determined and new information comes to light on previously classified enzymes. Presently (June 2006), 3927 EC numbers correspond to a defined unambiguous activity encoded by a protein. Note that IntEnz, the integrated relational enzyme database [2], now provides easy access to updated and curated data of the NC-IUBMB [1].

**Table 1:** Browsing the EC hierachy. For each level are indicated the total number of EC numbers and that of orphan EC numbers between brackets.

**Class**	**3 **1065 [336]													
**Subclass**	**3.1 **267 [113]	**3.2 **163 [56]		**3.3 **10 [4]	**3.4 **317 [49]	**3.5 **171 [70]	**3.6 **109 [36]	**3.7 **10 [4]	**3.8 **10 [1]	**3.9 **1 [1]	**3.10 **2 [1]	**3.11 **2 [0]	**3.12 **1 [1]	**3.13 **2 [0]
**Sub-subclass**		**3.2.1 **140 [45]	**3.2.2 **23 [11]											

Unexpectedly, Peter Karp [3] and us [4,5] independently observed that a significant part of these curated and approved EC numbers does not correspond to any amino acid sequence in public databases. Recent updates of our previous results confirm this very large gap between known enzyme function and recorded protein sequence. There are presently only 2483 EC numbers having at least one associated sequence in the release 8.1 (13-Jun-2006) of the UniProt Knowledgebase [6]. We have used the term orphan enzyme activities [4] for the 1444 EC numbers that do not have a sequence associated with them. Remarkably, these orphan enzyme activities currently represent 36.8% of the 3927 retained EC numbers.

We have already shown that orphans are present at about the same proportion in every class and subclass of enzyme activities [4]. Likewise, we found no correlation between orphan distribution and main functional categories. 25.3% of the enzyme activities involved in well-studied metabolic pathways are sequence-less while we found 49.5% orphans among non-metabolic enzyme activities [4].

Thus, it appears that there is an important gap between function and sequence, which implies that its progressive bridging would require a concerted effort as already underlined [3,4]. Accordingly, we have built ORENZA, a database of ORphan ENZyme Activities, to offer such a tool to the research community. Hereafter, we describe the content of this resource and we detail how to use it in order to reach the goals defined above.

## Construction and content

### Structure of the ORENZA database

In order to build an efficient relational database that will help to identify the encoding gene for the maximum number of sequence-less enzyme activities (the so-called orphan enzymes [4]) we have retrieved data from various public databases and we have organized them as described below.

### Data collection

**Figure 1:**
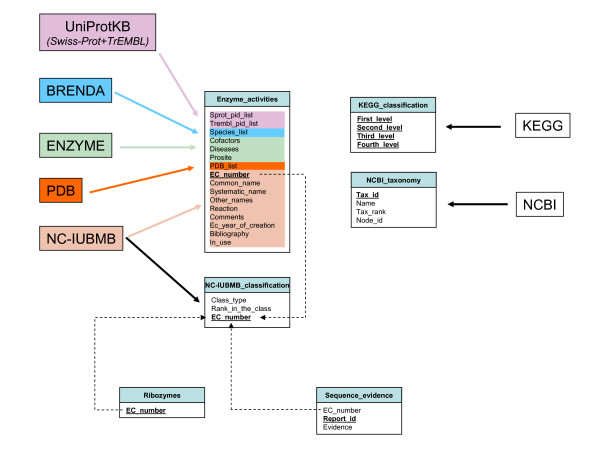
**Schema of the ORENZA relational database**. The primary key of each table is in bold underlined type. Dashed arrows indicate references to foreign keys. Plain arrows represent the origin of the data stored in each table. Moreover, for the table Enzyme_activities the origin of the data is indicated by the same color code used to identify each of the following major primary databases used in our analysis: UniProt (purple), BRENDA (blue), ENZYME (green), PDB (orange) and NC-IUBMB (beige).

There are two primary sources of information about each enzyme, corresponding respectively to all data about its activity (EC number), namely the Enzyme Nomenclature [1,2] and amino acid sequences as recorded in UniProt Knowledgebase [6]. Fig. 1 shows the different fields that were collected from both these sources and how they are organized in one main table. Moreover, we added additional – but highly important – features about each enzyme such as its role in metabolism (data recovered from KEGG [7]), the names of the organism(s) where it has been studied (data extracted from BRENDA [8] and from UniProt [6]), and the taxonomy of these organisms (data extracted from the NCBI [9]), and various pieces of information extracted from ENZYME [10] such as cofactors, possible role in disease and motifs found in PROSITE [11]. These secondary characteristics are confined to small tables or added directly to the main one as in the case of the 3D structure (data recovered from PDB [12]). We wrote Perl scripts in order to extract and periodically update the relevant information from the following public resources: NC-IUBMB, ENZYME, KEGG, BRENDA, UNIPROT, and PDB. Note also the addition of a couple of other tables, one is listing ribozymes (only one, presently), the other one listing the individual contributions made by external experts on their sequence data (see below for more details).

### Checking orphanity

A Perl script screened the occurrence of EC numbers in UniProt Knowledgebase [6]. Any EC number assigned by the NC-IUBMB [1] that is not referenced in UniProt is defined as an orphan enzyme activity. Note that we did not take into account partial or incomplete EC numbers (318 in the present version of UniProt) but too ambiguous [13] for sound use.

### Structuring the relational database and implementing the web resource

We chose to use exclusively open source tools to build ORENZA database.

Accordingly, PostgreSQL 8.1 [14], one of the most advanced open source databases, was installed on a Linux platform. PHP language [15] was used to structure the Web service and to better exploit the queries from the relational database.

## Utility

### Browsing and searching ORENZA

One can browse and/or search ORENZA using three main avenues as described in detail below.

#### Browsing the whole set of EC numbers

**Figure 2:**
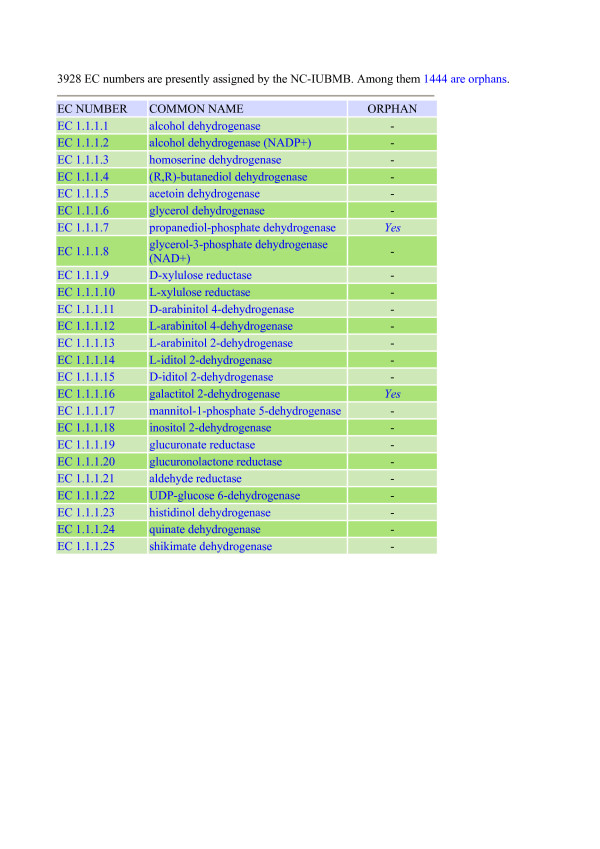
**Extract from the full list of enzymes classified by the NC-IUBMB, along with their associated orphanity**. For each line EC number, common name and orphanity are indicated. The total number of enzymes and the total number of orphan enzymes activities are indicated on top.

**Figure 3:**
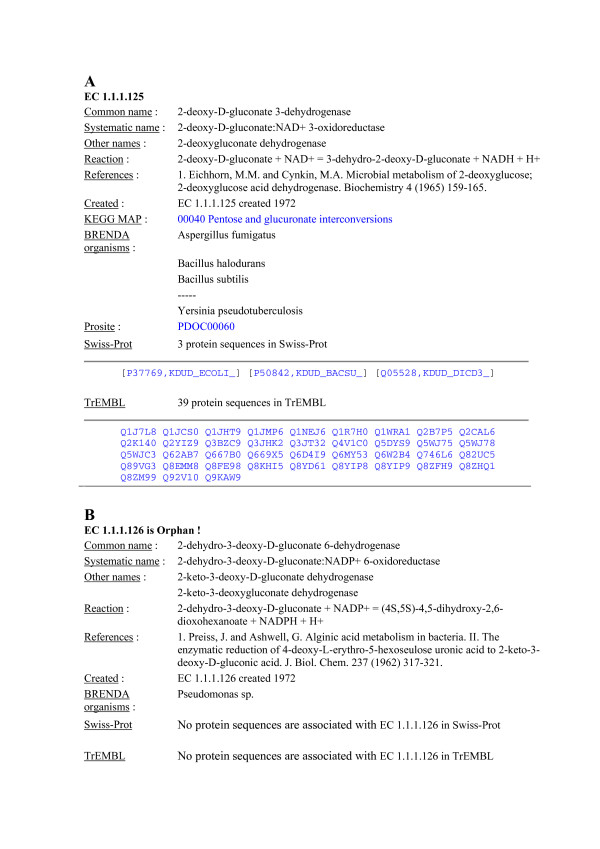
**Details of specific enzymes**. 3A: example of an enzyme entry with associated amino acid sequences. 3B: example of an orphan EC number. The fact that the enzyme is an orphan enzyme is noted after the EC number and in the Swiss-Prot and TrEMBL fields.

The complete list of EC numbers is directly available by a simple click. It corresponds to the most recent version of NC-IUBMB [1]. The obtained view displays the list as a three-column table where each line corresponds to a specific EC number, the common name of the corresponding enzyme and a computed annotation about its possible orphanity, respectively (Fig. 2). Note also that the upper line of this view shows a summary indicating the total number of the EC numbers present in the selection (including the ribozyme) as well as that of the orphan EC numbers, respectively. The entire list, which can be easily downloaded as a text file, is completely dynamic. A click on a line opens a new view delivering a wealth of information about the selected EC number that is structured in three successive levels. Fig. 3A shows an example in the case of EC 1.1.1.125 with notification of many features.

The first level consists of characteristics of the enzymatic activity and its history. The description section contains information taken from the NC-IUBMB data such as the different names (common, systematic, and others) of the enzyme, a scheme of the reaction(s) it catalyses and other data about the cofactors and NC-IUBMB comments about the reaction that are extracted from the ENZYME database [10]. In the history part, we list fundamental references, and the date of creation of the entry in the official NC-IUBMB nomenclature.

The second level presents information about the position of the enzyme in the cell metabolism with the corresponding number of a KEGG map [7], and its taxonomic ubiquity with a list of organisms where this enzymatic activity has been characterized as recorded in the BRENDA database [8].

The third level exhibits information about the peptidic molecule such as motifs (from PROSITE [11]), the lists of amino acid sequences found in SwissProt and TrEMBL, respectively [6]. If there is no sequence, as is the case for EC 1.1.1.126, which is labeled "orphan", this is clearly mentioned (Fig. 3B).

#### Browsing the orphan EC numbers

The second main avenue offered by ORENZA to explore the enzyme universe is the entire list, periodically updated, of the orphan enzyme activities. As described above, there are several ways to retrieve these orphans besides browsing the list in its entirety.

First, one can browse the different levels (class, subclass, etc.) of the EC hierarchy exactly as already described for the whole dataset of EC numbers.

**Figure 4:**
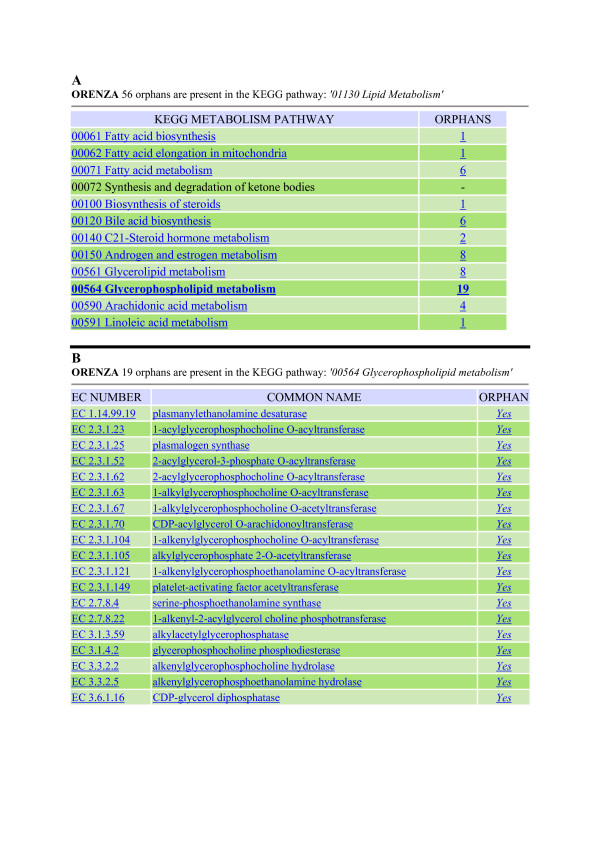
**List of orphan enzyme activities for various KEGG pathways**. 4A: List for pathway *'01130 Lipid Metabolism'*, sorted by sub-pathways. 4B: List of orphan EC numbers for pathway *'00564 Glycerophospholipid metabolism*.'

A second approach is to explore the metabolism hierarchy proposed by KEGG. For instance, clicking on Lipid Metabolism (56 orphans out of 246) opens a view showing the distribution of these orphans inside the 12 corresponding pathways (Fig. 4A). Among these 12 pathways, glycerophospholipid metabolism appears to have the most orphans (19). Another click unveils the full list of these enzyme activities involved in glycerophospholipid metabolism for which no amino acid sequence is available (Fig. 4B). Again, one can explore each enzyme in detail and copy/paste the corresponding information to save it as a text file.

**Figure 5:**
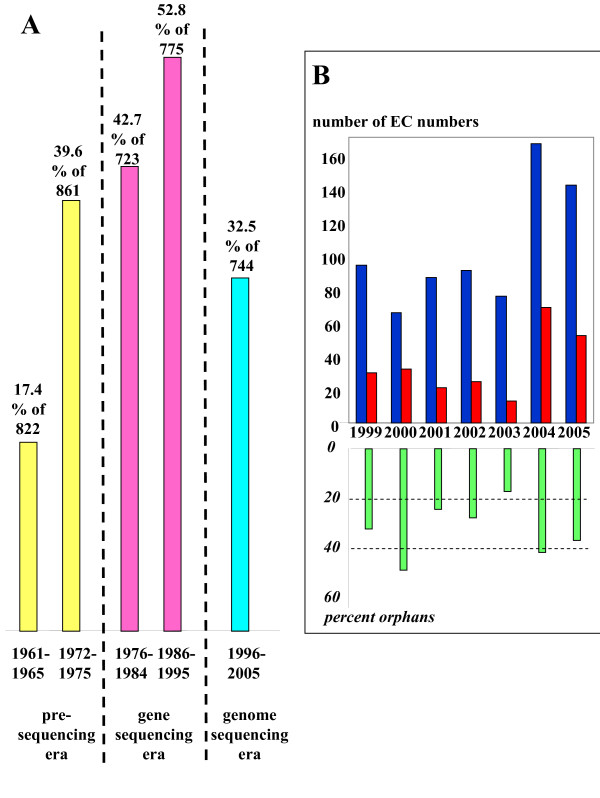
**Distribution of the creation year of orphan enzyme activities**. 5A. Distribution during the pre-sequencing era (yellow), the gene sequencing era (pink) and the genome sequencing era (cyan). 5B Number of enzymes created within the past seven years that have/lack sequence data. Total number of EC numbers is in blue, total number of orphan EC numbers in red and percentage of orphans in green.

A third way to browse the orphan EC numbers is to sort them by their year of creation. This permits one to observe that the relative proportion of orphans is independent of the progress of genome sequencing. Fig. 5A shows that many orphans appeared during the period of gene sequencing and that the level remained unexpectedly high during the present era of heavy genome sequencing. Fig. 5B zooms in on the last seven years and confirms this trend with a high proportion of orphans in 2000, 2004 and 2005.

**Figure 6:**
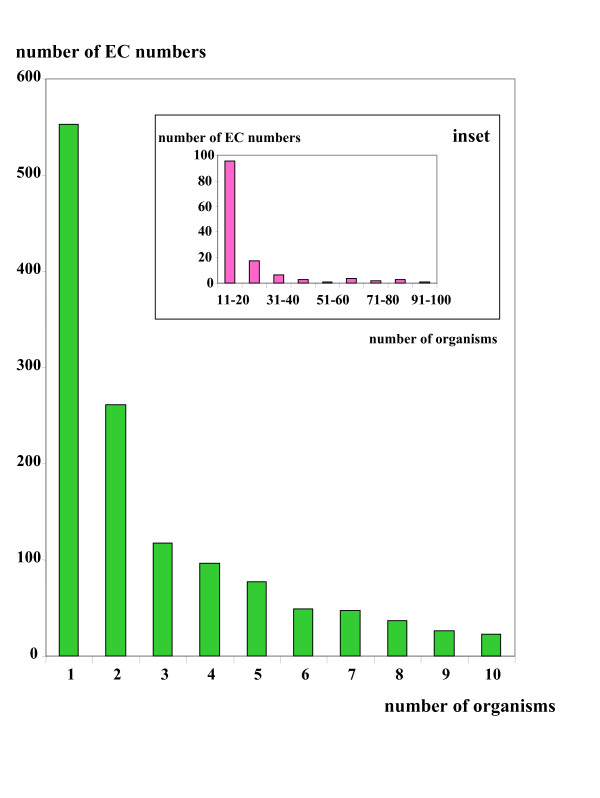
**Taxonomic distribution of orphan enzyme activities**. Green bars correspond to the distribution of the number of organisms (ranging from one to ten) where orphan EC numbers have been experimentally identified. In the inset, pink bars correspond to the number of orphan EC numbers identified in various ranges of number of organisms larger than ten organisms.

A fourth way to explore orphan enzyme activities is based on their occurrence in different organisms. Here, we access the entire list of orphan enzyme activities sorted by the number of organisms where these activities have been detected and experimentally studied. Beside the 39 EC numbers for which there is no information in the BRENDA resource, we find that a large majority (1286) of orphans is found in a limited number (1 to 10) of species (Fig. 6) but a few ones (132) have been found to have a large taxonomic distribution (Fig. 6, inset).

### Searching ORENZA

It is possible to query ORENZA for a specific enzyme activity by entering either the EC number or the enzyme name. For example, entering the word "aspartate" recovers 41 EC numbers, 13 being presently not assigned to a sequence.

Another interesting feature is the possibility of searching by species. For instance, entering the phrase "*Homo sapiens*" retrieves 1560 EC numbers that are present in human cells. Looking at the obtained list shows again a significant number of 225 orphans. The same observation is true for four other model organisms as shown in Table 2.

**Table 2:** Distribution of orphan enzyme activities in a few model organisms

	**EC numbers**		
**Model organisms**	**Total**	**Orphans (/total)**	**Species specific orphans (/total orphans)**
***Escherichia coli***	1792	189 (0.11)	25 (0.13)
***Arabidopsis thaliana***	651	22 (0.03)	0 (0)
***Saccharomyces cerevisiae***	1254	129 (0.10)	14 (0.11)
***Drosophila melanogaster***	417	16 (0.04)	4 (0.25)
***Homo sapiens***	1560	225 (0.14)	6 (0.02)

Interestingly, the proportion of orphans that are common to these five model organisms is extremely low. Only three EC numbers are found as orphans in the five organisms: EC 3.6.1.18 (FAD diphosphatase), EC 3.6.4.4 (plus-end-directed kinesin ATPase), and EC 3.6.4.5 (minus-end-directed kinesin ATPase). Moreover, only three EC numbers are found as orphans in *E. coli*, fungi and animals but not in plants: EC 1.1.1.43 (phosphogluconate 2-dehydrogenase), EC 1.5.3.2 (N-methyl-L-amino-acid oxidase), and EC 3.6.4.1 (myosin ATPase).

On the other hand, we have a few species-specific orphans as shown further on Table 2. For instance, six orphan EC numbers are reported uniquely in human cells (listed in Table 3) but the corresponding figures are as high as 25 for *E. coli *and 14 for *S. cerevisiae*, two organisms that have been intensively studied at the biochemical level for 60 years by thousands of laboratories worldwide.

**Table 3:** The six orphan enzyme activities that are specific to *Homo sapiens*.

**EC number**	**Enzyme name**	**role in human physiology**
EC 2.3.1.125	1-alkyl-2-acetylglycerol O-acyltransferase	platelet activation
EC 3.1.6.15	N-sulfoglucosamine-3-sulfatase	urinary infection by *Flavobacterium heparinum*
EC 1.1.1.160	dihydrobunolol dehydrogenase	liver physiology
EC 2.4.1.153	dolichyl-phosphate α-N-acetylglucosaminyltransferase	liver physiology
EC 3.1.2.13	S-succinylglutathione hydrolase	liver physiology
EC 5.1.3.19	chondroitin-glucuronate 5-epimerase	blood coagulation, cardiovascular disease, carcinogenesis

### Building an ORENZA community

We clearly need the help of a large array of experts to identify the putative sequence(s) associated with orphan enzyme activities [3,4]. In order to encourage such a collective effort, we propose, as a part of this ORENZA resource, a friendly tool that will allow people having sound knowledge about specific enzyme activities to make helpful suggestions. Moreover, such a resource could help to establish fruitful and dynamic interactions between different experts interested in the same field. Indeed, each suggestion (with identification of its author) will appear on ORENZA resource as a new item on each EC number's individual files. If several experts agree on the same suggestion, it would be transmitted to the curators of UniProt with a high degree of confidence. In cases where experts provide conflicting advice, all versions of the advice provided will be published as they have been set and validated. This would allow the community to decide, eventually.

## Discussion

The presence of so many EC numbers that do not have an associated sequence appears rather extraordinary at a time where we are inundated by genomic data. Such a situation is encroaching Research at different levels. Alleviating this problem would be very helpful for the difficult task of annotating and/or reannotating genomes. Thus, there is an urgent need to bridge this unwanted gap between biochemical knowledge and massive identification of coding sequences and we and others (see Karp [3]) think that the whole community must contribute to this task. This is why we built this ORENZA resource.

We designed this database to be an interactive tool allowing each expert to exploit his/her knowledge about an (or a group of related) enzyme(s) that have been registered as being an orphan enzyme activity.

Different cases may exist and we already described three of them where personal expertise would eliminate many errors and/or neglected instances. (i) A trivial error takes place when the enzyme has been correctly described in a sequence database but its EC number is not indicated. This is the case for example of glyceraldehyde 3-phosphate dehydrogenases as already shown [5]. One of these sequences (GAPOR, EC 1.2.7.6) has been entered in UniProt without its EC number although the information was given in relevant published papers. Presently, we estimate that up to 20% of the so-called orphan EC numbers might correspond to such a trivial incomplete annotation in the sequence databases (OL & BL, unpublished results). (ii) A sequence or a partial sequence has been previously determined but has not been published. We recently described such an instance in the case of putrescine carbamoyltransferases [16]. (iii) We further observed that around 50% of the present orphan EC numbers are found in only one species or a few closely related organisms as shown on Fig. 6. This is due, in the large majority of the cases, to the fact that we miss genetic tools for such imperfectly studied organisms. Moreover, the availability of genomic sequences for closely related species is useless when the orphan EC numbers are specific for the studied organisms (see Tables 2 and 3).

## Conclusion

We consider ORENZA to be a useful resource for all categories of biologists. Let us take for instance the data summarized in Table 2 and more precisely the observation that human cells harbour six enzyme activities that are not found elsewhere and that are not associated with any amino acid sequence (Table 3).

Any biologist would attempt to better understand the origin of such metabolic specificities. Any progress in this field could have positive consequences in terms of medical advances (see Table 3).

The genomicist would wonder if the occurrence of these six orphans is not an indicator of a big annotation problem in the current analysis of the human genome. The expert for either a specific enzyme or a physiological aspect related with these orphan enzyme activities would feel personally concerned and we hope that he/she will promptly answer such a challenge.

## Availability and requirements

ORENZA resource is freely available via the Internet at . The web accessibility has been tested to work with the Mozilla 1.7.12, Mozilla Firefox 1.5, and Internet Explorer 6.0 web browsers.

Complete lists of all EC numbers and of orphan EC numbers are available and will be periodically updated. All data can be easily downloaded as text files.

## Authors' contributions

OL wrote the different programs necessary to collect all data from public sources and to build the relational database and the web server. Both authors participated in the data analysis and wrote the paper.
